# AI Is Changing the Landscape of Academic Writing: What Can Be Done? Authors’ Reply to: AI Increases the Pressure to Overhaul the Scientific Peer Review Process. Comment on “Artificial Intelligence Can Generate Fraudulent but Authentic-Looking Scientific Medical Articles: Pandora’s Box Has Been Opened”

**DOI:** 10.2196/50844

**Published:** 2023-08-31

**Authors:** Martin Májovský, Tomas Mikolov, David Netuka

**Affiliations:** 1 Department of Neurosurgery and Neurooncology First Faculty of Medicine Charles University Prague Czech Republic; 2 Czech Institute of Informatics, Robotics and Cybernetics Czech Technical University in Prague Prague Czech Republic

**Keywords:** artificial intelligence, AI, publications, ethics, neurosurgery, ChatGPT, Chat Generative Pre-trained Transformer, language models, fraudulent medical articles

With every new technology, there are benefits that come hand in hand with the potential for misuse. Currently, many researchers are already using large language models (LLMs) for tasks such as language editing and abstract generation, which is beneficial and time-saving. It is undeniable that in the near future, we will witness dedicated tools that facilitate the preparation of scientific manuscripts, including tasks like reviewing relevant literature and generating discussion sections. However, an obvious danger arises: the possibility of fabricating entire articles using LLMs without conducting any genuine research [1]. It is important to note that LLMs do not enable scientists to cheat; scientific fraud has existed long before their advent, but LLMs simply make it easier.

As mentioned by Liu and Brown [2], the scientific community is under increasing pressure to overhaul the peer review and publishing processes. Solving complex problems is never a simple task. Should the use of LLMs be banned, or should we focus on detecting artificial intelligence (AI)–generated text? Banning the use of LLMs is a naive approach. Any AI-based tool designed to detect AI-generated text will inevitably fail because one can always train the next language model on the outputs of such a tool. So, what can be done? We believe that implementing the following measures, some of which are already in use, may help reduce the number of fraudulent papers generated by AI:

- *Provision of source data sets publicly*. Authors should provide anonymized data sets with all subject information with the submission as a supplementary material. This measure can discourage researchers from submitting completely fraudulent papers.
- *A meticulous review process*. Respectable publishers should emphasize a quality review process by selecting and educating high-profile reviewers. Some kind of reviewer award system may boost the motivation of potential reviewers. In addition to the award system, a reviewer-ranking system could be implemented based on the quality of the review reports.
- *Strict ethical regulations at the level of publishers*. Questions related to the use of LLMs should be incorporated into the publishing ethics questionnaire.
- *Strict ethical regulations at the level of academic institutions*. Researchers should be motivated at the level of their institutions (eg, universities) to publish high-quality, genuine research in esteemed journals.
- *Penalties for researchers who commit ethical misconduct*. Researchers who are convicted of ethical fraud should be penalized. This could be carried out by a temporary or permanent ban from publishing with certain publishers, limiting indexing, etc.

LLMs are dramatically transforming the landscape of academic writing. The scientific community should not resist the modern advances of generative AI but rather seek to accommodate them. Undoubtedly, this process will be tedious and challenging. We are pleased that our work [1] contributes to the important debate taking place across the scientific community regarding this topic.

## Abbreviations

AI: artificial intelligence
LLM: large language model
